# CA1 *Nampt* knockdown recapitulates hippocampal cognitive phenotypes in old mice which nicotinamide mononucleotide improves

**DOI:** 10.1038/s41514-018-0029-z

**Published:** 2018-11-08

**Authors:** Sean Johnson, David F. Wozniak, S. Imai

**Affiliations:** 1 Department of Developmental Biology, Washington University School of Medicine, St. Louis, MO 63110 USA; 2 Department of Psychiatry, The Taylor Family Institute for Innovative Psychiatric Research, Washington University School of Medicine, St. Louis, MO 63110 USA; 3 Present Address: Department of Gerontology, Laboratory of Molecular Life Science, Institute of Biomedical Research and Innovation, Kobe, Japan

## Abstract

Cognitive dysfunction is one of the most concerning outcomes in global population aging. However, the mechanisms by which cognitive functions are impaired during aging remain elusive. It has been established that NAD^+^ levels are reduced in multiple tissues and organs, including the brain. We found that NAD^+^ levels declined in the hippocampus of mice during the course of aging, and whereas we observed minimal age-related effects on spatial learning/memory capabilities in old mice, we discovered that they developed cognitive hypersensitivity in response to aversive stimulation during contextual fear conditioning tests. This cognitive hypersensitivity appears to be associated with alterations in emotionality (fear/anxiety) and sensory processing (shock sensitivity), rather than reflect genuine conditioning/retention effects, during aging. Supplementation of nicotinamide mononucleotide (NMN) improved the sensory processing aspect of the hypersensitivity and possibly other related behaviors. Specific knockdown of nicotinamide phosphoribosyltransferase (*Nampt*) in the CA1 region, but not in the dentate gyrus, recapitulates this cognitive hypersensitivity observed in old mice. We identified calcium/calmodulin-dependent serine protein kinase (*Cask*) as a potential downstream effector in response to age-associated NAD^+^ reduction in the hippocampus. *Cask* expression is responsive to NAD^+^ changes and also reduced in the hippocampus during aging. Short-term NMN supplementation can enhance *Cask* expression in the hippocampus of old mice. Its promoter activity is regulated in a Sirt1-dependent manner. Taken together, NAD^+^ reduction in the CA1 region contributes to development of age-associated cognitive dysfunction, aspects of which may be prevented or treated by enhancing NAD^+^ availability through supplementation of NAD^+^ intermediates, such as NMN.

## NAD+ and cognitive hypersensitivity during aging

Cognitive dysfunction is one of the most concerning outcomes in global population aging. However, the mechanisms of cognitive impairment during aging remain elusive. We found that in old mice, levels of nicotinamide adenine dinucleotide (NAD^+^), an essential chemical for all living organisms, declined in the hippocampus, a critical part of the brain for memory and learning. We also found that age-associated hypersensitivity in cognitive and behavioral functions (cognitive hypersensitivity) was induced by reduced NAD^+^ availability in the hippocampus. Supplementation of nicotinamide mononucleotide (NMN), a critical chemical that is converted to NAD^+^, is able to mitigate the cognitive hypersensitivity observed in old mice. Our findings provide new insights into how NAD^+^ decline affects age-associated anxiety/depression and how such impairments can be prevented or treated by enhancing NAD^+^.

## Introduction

Population aging is a topic of great concern in many countries worldwide. In the United States alone, the population of individuals aged 65 years or older is expected to reach almost 83 million by 2050, more than 20% of the population.^1^ Aging is a multitude of physiological functional decline, causing a loss of robustness in a variety of tissues and organs and culminating increased vulnerability to various insults and susceptibility to many different diseases. The central nervous system is not immune to the effects of aging. Cognitive impairment occurs in 22% of people over age 71 years in the United States.^2^ Equally as prevalent are mental disorders such as anxiety disorders that account for 10–20% of older adults and make them more common than either dementias or major depressive disorders.^3^ Of those with anxiety disorders, 90% are considered to be generalized anxiety disorder (GAD) or specific phobia, and GAD accounts for 50% of these cases.^4,5^ Late-life anxiety disorders place significant financial burden not only on individuals but also on the healthcare system as a whole. With the increasing aging population, resolutions to address these problems and offer meaningful benefit and improvement in the quality of life have become an ever more important issue.

It has been becoming a consensus that maintenance of nicotinamide adenine dinucleotide (NAD^+^), a classical coenzyme for redox reactions and a substrate for NAD^+^-consuming enzymes, is vital for the robust functionality of multiple tissues and organs.^6–8^ During the course of aging, however, levels of NAD^+^ in multiple peripheral tissues and in the brain, particularly in the hippocampus, decline significantly.^6^ This systemic decrease in NAD^+^ levels during aging is partly due to the decrease in nicotinamide phosphoribosyltransferase (NAMPT), the rate-limiting enzyme in a major NAD^+^ biosynthetic pathway in mammals.^6–8^ There are five major precursors used to synthesize NAD^+^: tryptophan, nicotinamide and nicotinic acid (two forms of vitamin B_3_), nicotinamide riboside, and nicotinamide mononucleotide. Among them, nicotinamide is the major precursor for mammalian NAD^+^ biosynthesis and is converted to nicotinamide mononucleotide (NMN), a key NAD^+^ intermediate, by NAMPT. Nicotinamide/nicotinic acid mononucleotide adenylyltransferases (NMNATs) convert NMN into NAD^+^.^6–8^ Indeed, it has been demonstrated that 12-month-long supplementation of NMN can effectively mitigate a multitude of age-associated functional decline in regular chow-fed normal B6 mice,^9^ implicating a possible use of NMN as a preventive and therapeutic anti-aging intervention.

Many enzymes, including poly-ADP-ribose polymerases, sirtuins, and CD38/CD157 ectoenzymes, are dependent on the continuous supply of NAD^+^ throughout the body. Sirtuins are a class of NAD^+^-dependent deacetylases/deacylases which have central roles in integrating nutritional signals into various physiological responses. Sirtuins regulate a number of critical biological processes, including metabolism, stress response, DNA repair, chromatin remodeling, circadian rhythm, and aging.^10,11^ There are seven mammalian sirtuins, SIRT1–7, and several of them have been reported to play important roles in the mammalian brain. For example, SIRT1 has been demonstrated to regulate long-term potentiation and learning and memory.^12,13^ SIRT1 also promotes cognitive functions in mouse models of Alzheimer disease and Huntington disease.^14–16^ We have also demonstrated that both SIRT1 and SIRT2 are important to promote oligodendrocyte differentiation from neural stem/progenitor cells.^17^ Interestingly, both SIRT1 and SIRT2 have also been connected to depressive behaviors.^18,19^

Our previous studies have demonstrated that NAMPT in forebrain excitatory neurons is critical for cognitive and behavioral functions.^17,20^ In these studies, we used mice lacking *Nampt* in forebrain excitatory neurons (*CaMKIIαNampt*^−/−^ mice). Although these mice exhibit remarkable phenotypes that demonstrate the importance of NAMPT in hippocampal cognitive functions, it is difficult to know whether NAMPT-mediated NAD^+^ biosynthesis indeed contributes to age-associated changes in hippocampal cognitive functions. Therefore, we decided to assess differences in cognitive and behavioral functions between young and aged mice. Then, we examined whether any hippocampal region-specific *Nampt* knockdown could recapitulate the observed cognitive phenotypes in aged mice. Our findings in this study shed a new light on the importance of NAMPT-mediated NAD^+^ biosynthesis in age-associated decline in hippocampal cognitive functions.

## Results

### NMN supplementation improves cognitive hypersensitivity in old mice

**Fig. 1:**
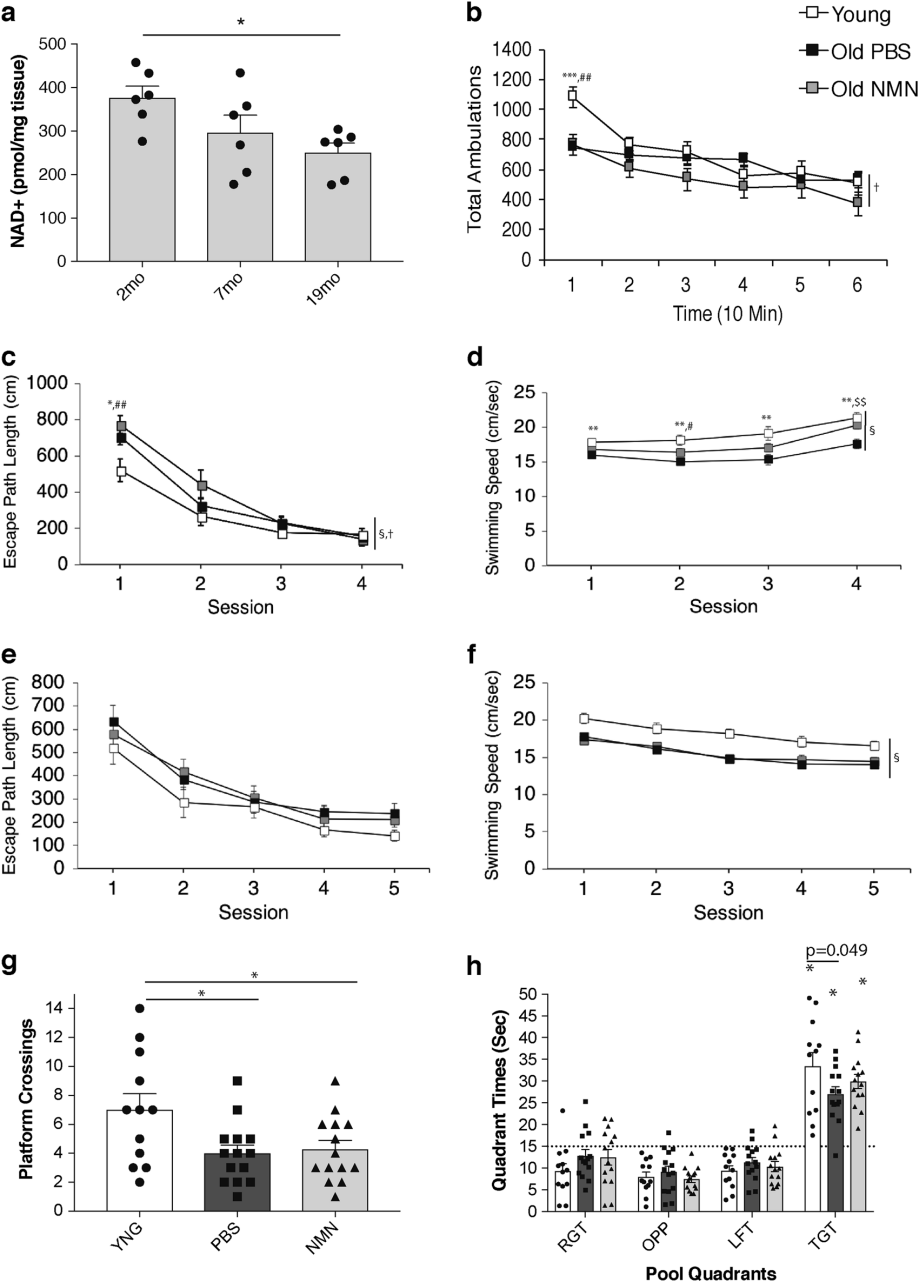
Old mice show significant decreases in hippocampal NAD^+^ levels but retain their spatial acquisition and learning capabilities. **a** Hippocampal NAD^+^ levels in male mice at 2, 7, and 19 months of age (*n* = 6; *p < 0.05, one-way ANOVA with LSD post hoc test). **b** Total ambulations were measured in the 1 h locomotor activity tests on PBS-treated 2-month-old (*n* = 12), PBS-treated 20-month-old (*n* = 14), and NMN-treated 20-month-old (*n* = 14) male mice. **c**–**h** Evaluation of PBS-treated 2-month-old (*n* = 12), PBS-treated 20-month-old (*n* = 14), and NMN-treated 20-month-old (*n* = 14) male mice in the MWM tests. Escape path lengths and swimming speeds during the cued (**c**, **d**) and place (**e**, **f**) conditions are shown. Platform crossings (**g**) and times spent in each quadrant (**h**) during the probe condition are also shown. Data are presented as mean ± SE. Statistical analyses were conducted by two-way repeated measures ANOVA for (**b–f**) and one-way ANOVA for (**a**, **g**, **h**). Significant group effects and group by time or sessions interactions are denoted by symbols at the right sides of the graphs (§ and †, respectively). **b**–**f** *p < 0.05, ^**, ##, $$^*p* < 0.01, ^***, ###^*p* < 0.001 (*, **, *** for PBS-treated young vs. PBS-treated old mice; ^#, ##^ for PBS-treated young vs. NMN-treated old mice; ^$$^ for PBS-treated old vs. NMN-treated old mice). g *p < 0.05 for the comparison to PBS-treated young mice. **h** Significant spatial bias toward the target quadrant is denoted (*)

It has been well established that NAD^+^ levels decline in multiple tissues and organs during the course of aging.^6^ In the hippocampus, we were able to confirm similar NAD^+^ decline (Fig. 1a). Comparing hippocampal NAD^+^ levels between 2, 7, and 19 months of age, we observed a gradual NAD^+^ decrease by ~40% (Fig. 1a). To examine whether any cognitive and behavioral impairments are associated with this NAD^+^ decrease, we set out a battery of cognitive and behavioral analyses for 2- and 20-month-old mice. Additionally, we decided to evaluate another cohort of 20-month-old mice to which NMN (300 mg/kg/day) was administered through oral gavage for ~3 weeks. It has been shown in many studies, including ours, that NMN supplementation can circumvent the reduction in NAD^+^ caused by lowered expression of NAMPT in old mice.^6^ Indeed, we confirmed that NMN administration was able to increase hippocampal NAD^+^ levels significantly in old mice (Supplementary Fig. 1).

The phosphate-buffered saline (PBS)-treated young, PBS-treated old, and NMN-treated old groups of mice were first evaluated on a 1-h locomotor activity test and then on a battery of sensorimotor measures. A repeated measures analysis of variance (rmANOVA) conducted on the total ambulations (whole body movements) data from the 1-h locomotor activity test showed a nonsignificant group effect, although there was a significant group by time interaction (Fig. 1b; *F*_(10, 185)_ = 4.31, *p* = 0.0001). A large component of the interaction effect was due to differences between the PBS-treated young and PBS-treated old groups during the first 10-min time point (*p* = 0.001), and between the PBS-treated young and NMN-treated old groups for the same time interval (*p* = 0.002). Analysis of the data from the sensorimotor battery indicated that the young mice generally performed better than the old groups, particularly on the platform, pole, and 60° inclined screen tests (Supplementary Fig. 2).

Spatial learning and memory capabilities of the mice were subsequently assessed in the Morris water maze (MWM). The escape path length data from the cued trials (visible platform; variable location) yielded a significant group effect (Fig. 1c; *F*_(2, 37)_ = 3.86, *p* = 0.030) and a significant group by session interaction (*F*_(6, 111)_ = 2.45, *p* = 0.038). This interaction effect was mostly due to significantly increased path lengths in the PBS-treated and NMN-treated old groups compared to the young group during the first session (*p* = 0.017 and *p* = 0.002, respectively). Although the two old groups showed impaired performance during initial exposure to the MWM, all three groups were performing at comparable levels by the end of the cued trials. A group effect was found with regard to swimming speeds during the cued trials (Fig. 1d; *F*_(2, 37)_ = 11.81, *p* = 0.0001). On average across training, the young mice swam significantly faster than the old mice in the PBS-treated and the NMN-treated groups (*p* < 0.00005 and *p* = 0.027, respectively), while the NMN-treated old mice swam faster than the PBS-treated old mice (*p* = 0.012). The magnitude of the effects appeared to be greatest between the PBS-treated young versus PBS-treated old mice since significant differences (beyond Bonferroni correction: *p* < 0.05/4 = 0.0125) were found for each of the four sessions (all *p* values < 0.0025), whereas large differences were only observed for session 2 (*p* = 0.038) between the young and NMN-treated old mice and for block 4 for the NMN-treated old versus the PBS-treated old groups (*p* = 0.006).

Analysis of the escape path length data for the place trials (hidden platform, single location; testing spatial learning) showed that there were no significant main or interaction effects involving the group variable, suggesting comparable levels of acquisition across the three groups (Fig. 1e). A significant effect of session (*F*_(4, 68)_ = 21.69, *p* < 0.00005) indicated that, in general, the groups showed significant improvement across sessions, suggesting that learning had occurred. Within-subject comparisons conducted for each group between session 1 versus session 5 also showed significantly improved performance for each group (all *p* values < 0.009). Similar to the results from the cued trials, significant differences in swimming speeds were observed during the place condition (Fig. 1f; group effect: *F*_(2, 17)_ = 5.11, *p* = 0.018), with results from simple main effect tests showing that the PBS-treated young mice swam faster than the PBS- and NMN-treated old groups (*p* = 0.007 and *p* = 0.027, respectively) on average across sessions, whereas the two old groups displayed similar swimming speeds (Fig. 1f). A significant group effect (*F*_(2, 37)_ = 4.40, *p* = 0.019) with regard to platform crossings during the probe trial (platform removed; testing retention) indicated that differences existed among groups in retention performance, with subsequent pair-wise comparisons showing that the PBS-treated young mice made more crossings over the former platform location than either the PBS-treated old (*p* = 0.010) or the NMN-treated old (*p* = 0.019) groups, while the older groups performed similarly (Fig. 1g). However, all groups showed spatial bias for the target quadrant (Fig. 1h). Also, there were trends for the young group to spend more time in the target quadrant compared to the old groups and for the NMN-treated group to spend more time in the target quadrant compared to the PBS-treated old mice (Fig. 1h).

**Fig. 2:**
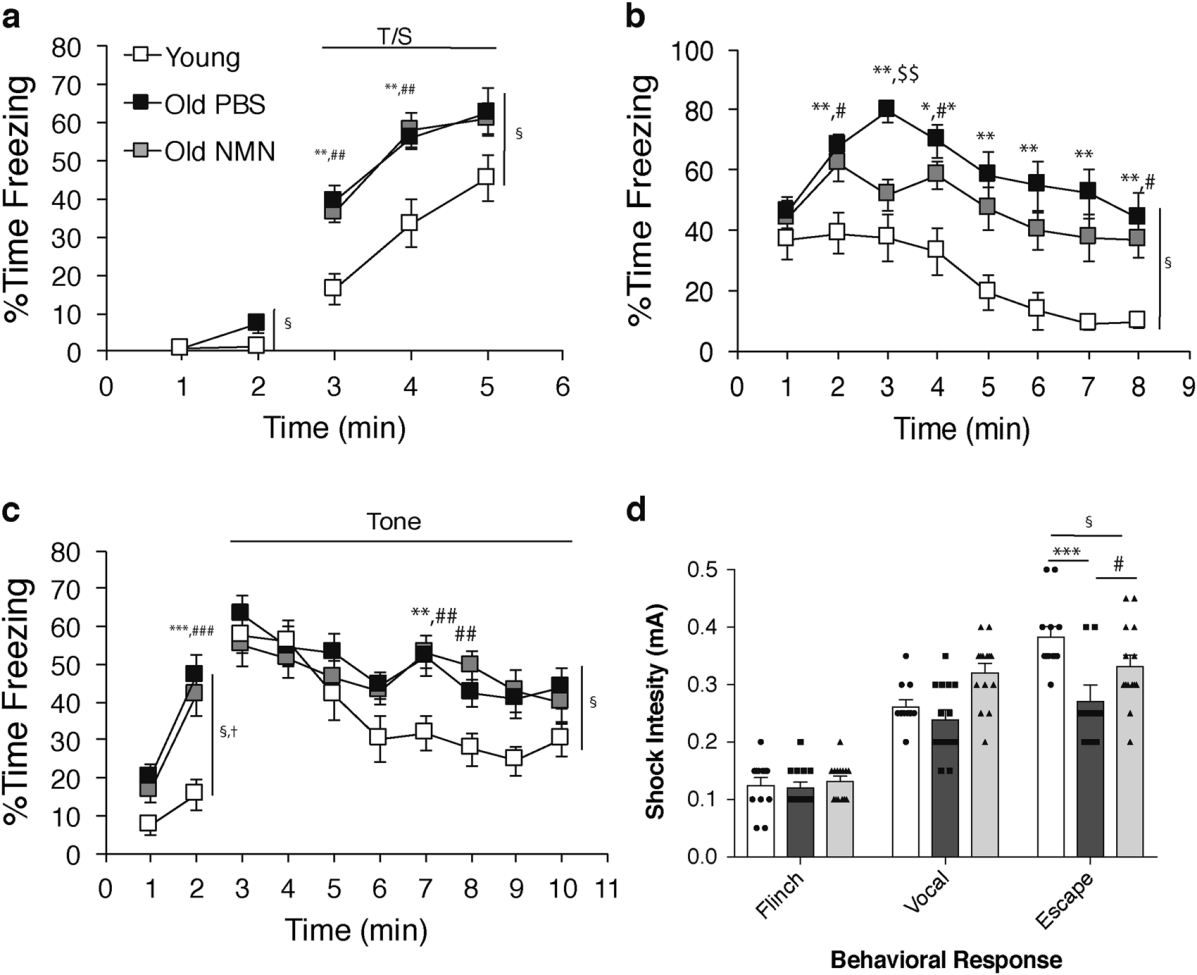
Old mice exhibit cognitive hypersensitivity, aspects of which are improved by NMN. **a**–**c** Contextual fear conditioning tests were conducted on PBS-treated 2-month-old (*n* = 12), PBS-treated 20-month-old (*n* = 14), and NMN-treated 20-month-old (*n* = 14) male mice. Values of percent time freezing are shown for baseline and tone-shock (CS-US) training periods on day 1 (**a**), contextual fear conditioning on day 2 (**b**), and the auditory cue period on day 3 (**c**). Statistical analyses were conducted by two-way repeated measures (rm) ANOVA, and differences at each time point were evaluated by one-way ANOVA. **d** Shock sensitivities for flinch response, vocalization, and escape response are shown for the same groups of mice used in (**a–c**). Data are presented as mean ± SE. Significant group effects and group by time interactions are denoted by symbols at the right sides of the graphs (§ and †, respectively); ^#^*p* < 0.05, ^**, ##^*p* < 0.01, ^***^, ^###^, ^$$$^*p* < 0.001 (**, *** for PBS-treated young vs. PBS-treated old mice; ^#, ##, ###^ for PBS-treated young vs. NMN-treated old mice; ^$$$^ for PBS-treated old vs. NMN-treated old mice)

Following completion of the MWM testing, the mice were then assessed on the conditioned fear procedure to evaluate nonspatial Pavlovian conditioning capabilities. Analysis of baseline freezing levels measured on day 1 (Fig. 2a) showed a nonsignificant group effect, but a significant group by time interaction (*F*_(2, 37)_ = 3.95, *p* = 0.028). All three groups showed comparable freezing levels during minute 1, and the PBS-treated and NMN-treated old groups exhibited higher freezing levels compared to the PBS-treated young mice during minute 2 (*p* = 0.029 and *p* = 0.032). These differences were not significant according to Bonferroni correction (*p* < 0.05/2 = 0.025) for the comparisons made at each minute. In contrast, there were large differences with regard to freezing levels in response to the tone-shock training on day 1, for which a significant group effect was observed (Fig. 2a; *F*_(2, 37)_ = 12.22, *p* = 0.0001). Subsequent simple main effect tests showed that the PBS-treated and NMN-treated old mice displayed significantly elevated freezing levels compared to the PBS-young mice on average across the 3 min of training (*p* = 0.0001 for each comparison) with significant differences being observed for minutes 3 and 4 (all *p* values < 0.002). The contextual fear data from day 2 yielded a robust group effect (Fig. 2b; *F*_(2, 37)_ = 15.74, *p* < 0.00005). Specifically, the PBS-treated and NMN-treated old mice had significantly increased freezing levels compared to the PBS-treated young mice on average across minutes of the test session (Fig. 2b; *p* < 0.00005 and *p* = 0.0008, respectively; see the legend for *p* values of individual time-related comparisons). In addition, a strong trend towards the NMN-treated old mice showing reduced freezing levels compared to the PBS-treated old mice (*p* = 0.052) was found, with a major component of this trend being due to a large difference between groups observed during minute 3 (*p* = 0.001). On day 3, a significant group effect (*F*_(2, 37)_ = 8.96, *p* = 0.0007), and group by time interaction (*F*_(2, 37)_ = 7.70, *p* = 0.002) were observed when the mice were placed into a different chamber containing new environmental cues (altered context baseline) (Fig. 2c). Subsequent contrasts showed that the PBS-treated old mice exhibited significantly greater levels of freezing compared to the young mice for minutes 1 and 2 (*p* = 0.005 and *p* = 0.0001, respectively) beyond Bonferroni correction (*p* = 0.05/2 = 0.025). Also, compared to the young group, the NMN-treated old mice showed significantly elevated levels of freezing for minute 2 (*p* = 0.001) and fairly large differences for minute 1 (*p* = 0.045), while no differences were observed between the two old groups. A significant group effect was also observed for the auditory cue test on day 3 (Fig. 2c; *F*_(2, 37)_ = 4.68, *p* = 0.015), with higher freezing levels again being observed between each of the PBS-treated and NMN-treated old groups versus the young group (*p* = 0.007 and 0.019), and also with the bulk of these effects being due to differences between the young and old groups during minutes 6–10 (see Fig. 2c for *p* values for individual comparisons). Freezing levels did not differ significantly between the two old groups during the auditory cue test.

In order to aid in the interpretation of the performance differences observed during the conditioned fear testing, the groups of mice were subsequently evaluated for their sensitivity to foot shock. Whereas nonsignificant group effects were found for flinching and vocalizing, a robust, significant group effect was found for escape behavior (Fig. 2d; one-way ANOVA, *F*_(2, 37)_ = 8.48, *p* = 0.0009). Results from a pair-wise comparison revealed that significantly lower levels of shock elicited escape behavior in the PBS-treated old mice compared to those observed in the PBS-treated young mice (*p* = 0.0002). Importantly, significantly higher levels of shock were required to elicit escape responses in the NMN-treated old mice compared to the PBS-treated old mice (*p* = 0.020). A similar trend was also observed for vocalizing. These results suggest that a heightened degree of sensitization is present in old mice, which reflects alterations in both sensory processing and emotionality (fear and/or anxiety). It is important to note that although no differences in freezing levels were observed among groups during the initial baseline period on day 1, each of the old groups displayed significantly higher levels of freezing during the altered context baseline period on day 3, compared to the young mice. This suggests that the age-related differences observed during the contextual fear test on day 2 were not due to differences in conditioning/retention, but rather to a heightened level of sensitization to the general experimental procedures following exposure to the foot shock in the two old groups. Thus, we propose to use the term cognitive hypersensitivity in characterizing age-related changes in sensory processing and emotionality, and these changes can be partially ameliorated by NMN supplementation in old mice.

### CA1-specific *Nampt* knockdown recapitulates age-associated hypersensitivity in conditioned fear response

Previous studies have shown that targeting *Nampt* either through inhibition of its enzymatic activity by FK866, a potent NAMPT inhibitor, or knockdown by small interfering RNA can dramatically reduce the intracellular content of NAD^+^.^17,21^ Given that both *Nampt* expression and NAD^+^ content decrease in the hippocampus during the course of aging, we hypothesized that the reduction in hippocampal NAD^+^ levels by reducing *Nampt* expression may recapitulate the cognitive phenotypes observed in old mice. We also hypothesized that a region-specific reduction in NAD^+^ levels might be important because the cognitive phenotypes of *CaMKIIαNampt*^−/−^ mice are very different from those of wild-type old mice.^22^

**Fig. 3:**
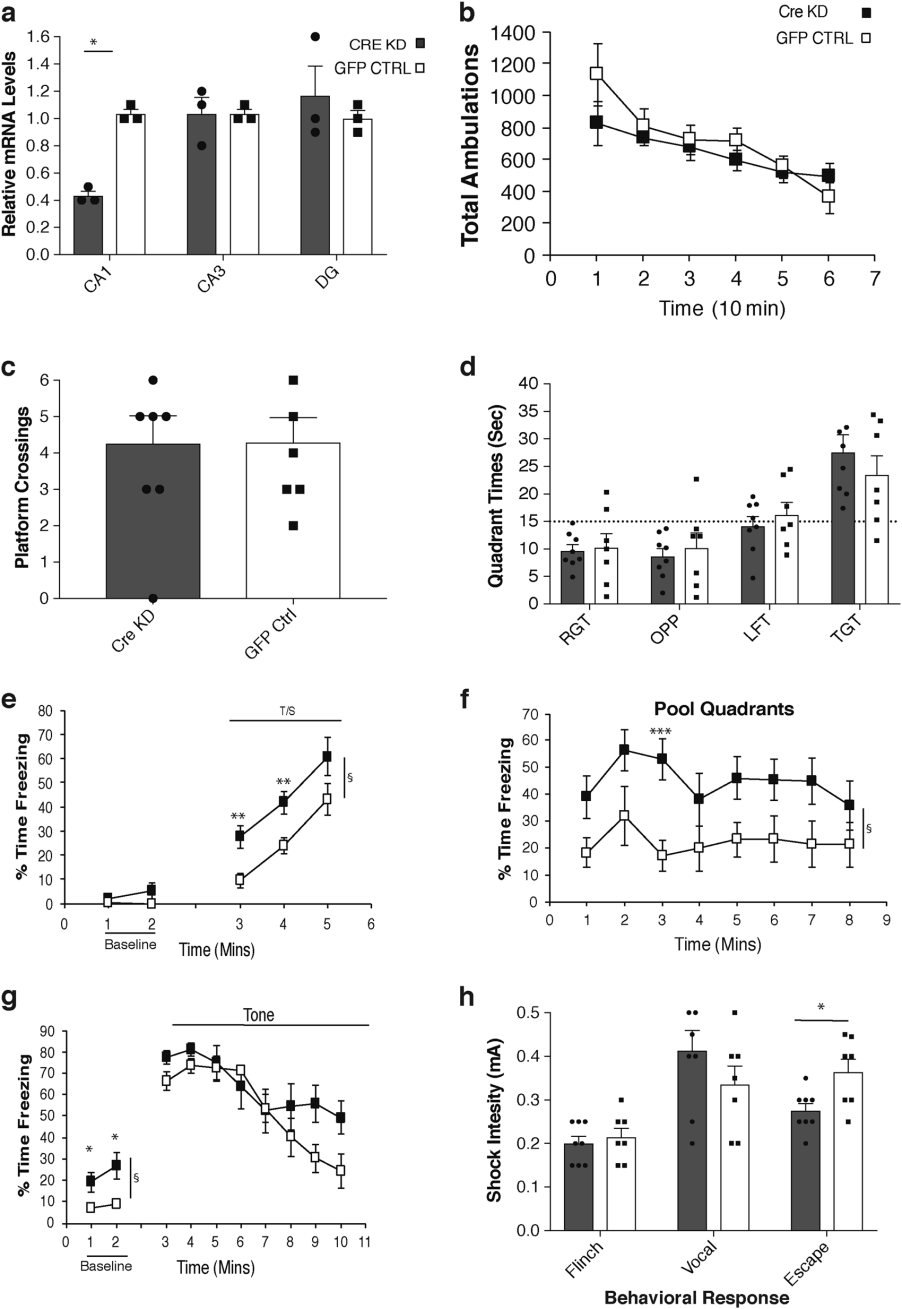
CA1-specific *Nampt* knockdown recapitulates cognitive hypersensitivity observed in old mice. **a** *Nampt* mRNA levels in the CA1, CA3, and DG regions of CA1-specific *Nampt* knockdown and GFP-injected control mice (*n* = 3 mice for each group; *p < 0.05, unpaired Student’s *t*-test). **b** Total ambulations were quantified during the 1 h locomotor activity test conducted on CA1-specific *Nampt* knockdown mice (*n* = 8) and GFP-injected control mice (*n* = 7). **c**, **d** Analysis of the MWM probe trial data did not reveal any significant differences between CA1-specific *Nampt* knockdown (*n* = 8) and GFP-injected control (*n* = 7) mice (unpaired Student's *t*-test, two-way rmANOVA). **e**–**g** Contextual fear conditioning tests were conducted on CA1-specific *Nampt* knockdown (*n* = 8) and GFP-injected control (*n* = 7) male mice. Values of percent time freezing are shown from the tone-shock (CS-US) training period on day 1 (**e**), contextual fear conditioning on day 2 (**f**), and the auditory cue period on day 3 (**g**). Statistical analyses were conducted by two-way rmANOVA, and significant group effects are denoted by a symbol (§) at the right sides of the graphs; *p < 0.05, **p < 0.01, ***p < 0.001. **h** Shock sensitivities for flinch response, vocalization, and escape response are shown for the same groups of mice used in (**e**–**g**) (*p < 0.05, unpaired Student’s *t*-test). Data are presented as mean ± SE

To address these hypotheses, we decided to create Cornu Ammonis 1 (CA1)-specific *Nampt* knockdown mice because the CA1 region is the most vulnerable to stresses and other neurodegenerative changes.^23–25^ Floxed *Nampt* mice at 3 months of age were injected stereotactically with adeno-associated viruses that carried Cre recombinase and green fluorescent protein (GFP) genes (Cre-GFP) or the GFP gene alone into the CA1 region of the hippocampus. The injected Cre-GFP virus was restricted to the dorsal CA1 region and showed ~60% knockdown of *Nampt* expression only in the CA1 region, but not in CA3 and dentate gyrus (DG) regions (Fig. 3a). At 4 weeks following injections, these CA1-specific *Nampt* knockdown (CA1*Nampt* KD) and GFP virus-injected control (GFP CON) mice were evaluated on the same set of behavioral tests. The two groups did not differ in general ambulatory activity with each exhibiting decreased levels over time reflecting habituation to the novel environment (Fig. 3b). The CA1*Nampt* KD and GFP CON mice also performed similarly on the battery of sensorimotor measures (data not shown). Additionally, no significant performance differences were observed between the two groups on the cued, place, or probe (Fig. 3c, d) trials during the MWM test.

During day 1 of the conditioned fear procedure, similar freezing levels between the CA1*Nampt* KD and GFP CON mice were observed during the baseline period, although the CA1*Nampt* KD mice did exhibit significantly increased freezing levels during tone-shock training (Fig. 3e; group effect: *F*_(1, 11)_ = 14.33, *p* = 0.003), with pair-wise comparisons showing significant differences (according to Bonferroni correction: *p* < 0.05/3 = 0.017) between the groups during minutes 3 (*p* = 0.008) and 4 (*p* = 0.005), and large differences during minute 5 (*p* = 0.043). Importantly, CA1*Nampt* KD mice displayed significantly increased freezing levels during the contextual fear test (Fig. 3f), relative to the GFP CON group (group effect: *F*_(1, 11)_ = 7.34, *p* = 0.020). Subsequent pair-wise comparisons revealed a significant difference (according to Bonferroni correction: *p* < 0.05/8 = 0.0063) between groups at 3 min (*p* = 0.0002), with large differences being observed at 1 min (*p* = 0.039) and 5 min (*p* = 0.019). Thus, the differences in freezing levels between CA1*Nampt* KD and GFP CON mice were similar to those observed between the young and old mice described above (Fig. 2b). Sex was included as a variable in the analyses since both males and females were used in this cohort, and significant sex effects were found for the tone-shock training and contextual fear test data (*p* = 0.004 and 0.005, respectively). However, sex was not observed to significantly interact with group in any way. The altered context baseline data on day 3 indicated that the CA1*Nampt* KD mice also showed increased levels of freezing relative to the GFP CON group when placed into the chamber containing new environmental cues (Fig. 3g; group effect: *F*_(1, 11)_ = 9.97, *p* = 0.009), with significant differences being observed during minutes 1 (*p* = 0.024) and 2 (*p* = 0.014). These results suggest that the differences between groups observed during the contextual fear test (day 2) were a function of heightened sensitization on the part of the CA1*Nampt* KD mice rather than true conditioning/retention effects. The auditory cue test on day 3 revealed nonsignificant effects for the group variable and the group by time interaction, although there was a trend toward significance for the interaction effect (*p* = 0.063), and there were trends towards differences in freezing levels between the CA1*Nampt* KD and GFP CON mice at 3 min (*p* = 0.049), 9 min (*p* = 0.057), and 10 min (*p* = 0.061). No significant sex effects were revealed from the analysis of the day 3 data. The behavioral responses to foot shock showed that the CA1*Nampt* KD mice displayed an increased sensitivity (lowered threshold) in escape behaviors (Fig. 3h; group effect: *F*_(1, 11)_ = 8.52, *p* = 0.014), whereas no significant group effects were observed for the other two shock-induced behavioral responses (Fig. 3h), nor were there any significant effects involving sex. Thus, the CA1*Nampt* KD mice showed a cognitive hypersensitivity compared to the GFP CON mice that was very similar to the age-related results from the tone-shock training, contextual fear test, and altered context baseline found between young and old mice.

**Fig. 4:**
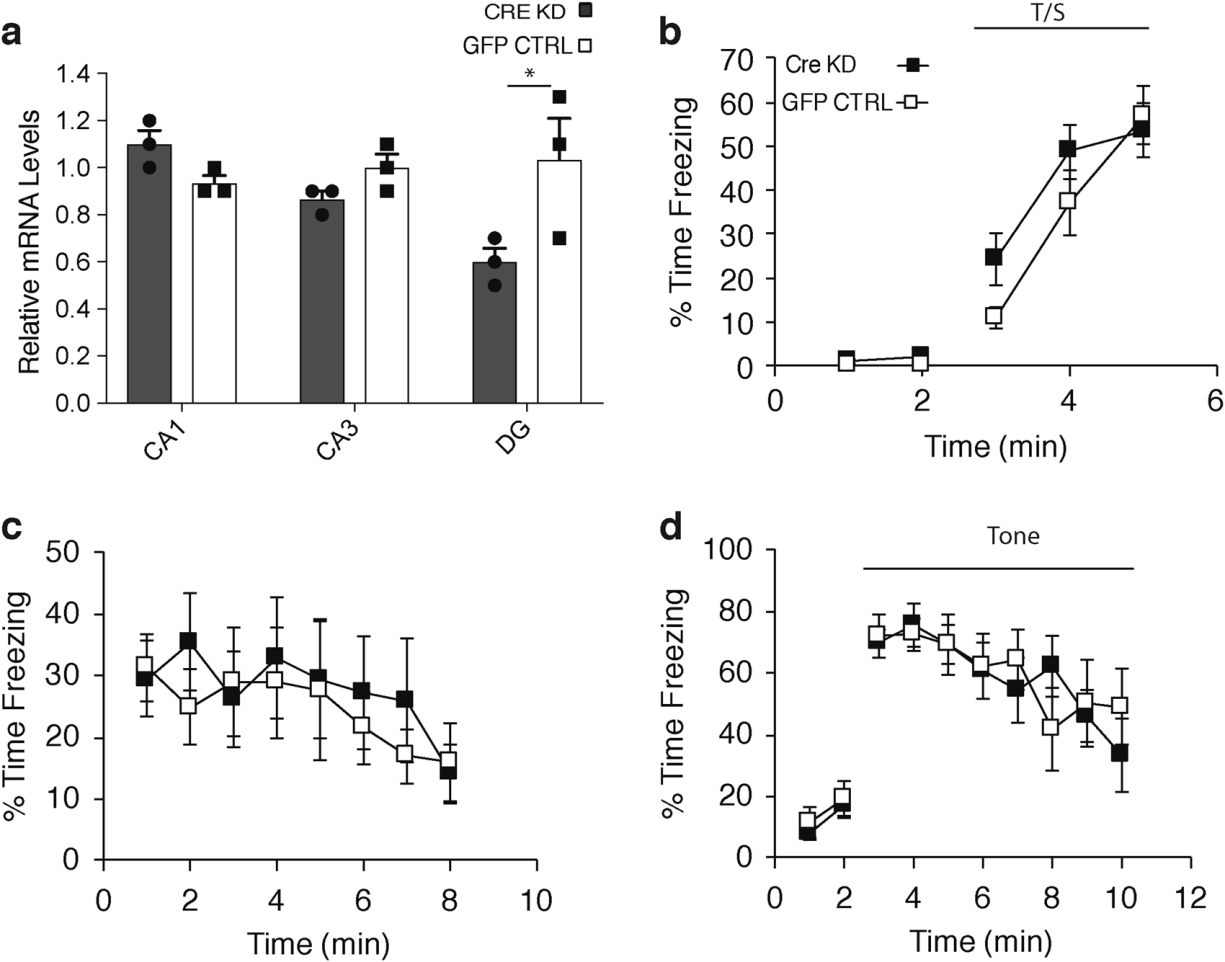
DG-specific *Nampt* knockdown mice do not show cognitive hypersensitivity. **a** *Nampt* mRNA levels in the CA1, CA3, and DG regions of DG-specific *Nampt* knockdown and GFP-injected control mice (*n* = 3 mice for each group; *p < 0.05, unpaired Student’s *t*-test). **b**–**d** Contextual fear conditioning tests were conducted on DG-specific *Nampt* knockdown (*n* = 7) and GFP-injected control (*n* = 6) mice. No significant main effects of group or group by time interactions were found from the analyses conducted for any of the components of the fear conditioning procedure. Values of percent time freezing are shown from the tone-shock (CS-US) training period on day 1 (**b**), contextual fear conditioning on day 2 (**c**), and the auditory cue period on day 3 (**d**). No statistical differences were detected by two-way rmANOVA. Data are presented as mean ± SE

To assess whether such cognitive hypersensitivity could be observed by knocking down *Nampt* in other hippocampal regions, we also created DG-specific *Nampt* knockdown mice. The *Nampt* knockdown efficiency was ~40% (Fig. 4a). These DG-specific *Nampt* knockdown mice displayed no cognitive hypersensitivity in contextual fear conditioning tests (Fig. 4b–d). These results suggest that CA1 plays an important role in developing age-associated cognitive hypersensitivity in old mice.

### Cask expression is NAD^+^ sensitive and reduced in aged hippocampi

**Fig. 5:**
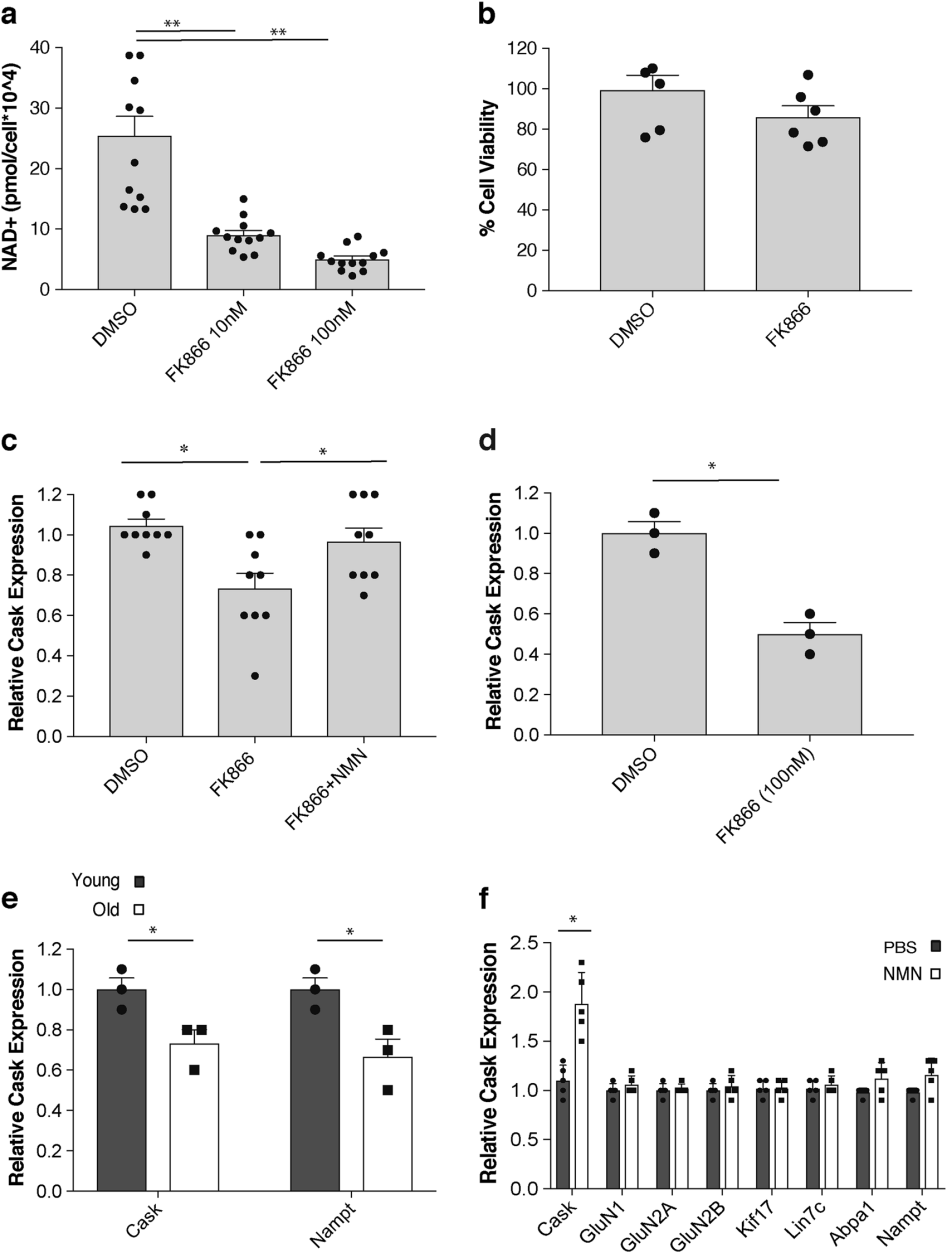
*Cask* expression is NAD^+^ sensitive and reduced in the hippocampus during aging. **a** Cellular NAD^+^ levels in primary hippocampal neurons treated with DMSO or FK866 at 10 nM and 100 nM for 48 h (*n* = 12; **p < 0.01, one-way ANOVA with LSD post hoc test). **b** Cell viability was assessed by measuring LDH in the supernatants from primary hippocampal neurons treated with DMSO or FK866 at 100 nM for 48 h (*n* = 6). **c** *Cask* mRNA levels in primary hippocampal neurons after treatment with FK866 (100 nM) or FK866 (100 nM) plus NMN (100 μM). Expression levels were normalized to those of DMSO-treated control cells (*n* = 9; *p < 0.05, one-way ANOVA with LSD post hoc test). **d** *Cask* mRNA levels in whole hippocampal *ex vivo* cultures after treatment with DMSO or FK866 (100 nM) (*n* = 3 for each condition; *p < 0.05, unpaired Student’s *t*-test). **e** mRNA levels of *Cask* and *Nampt* in hippocampi of 2-month-old (Y) and 20-month-old (O) mice (*n* = 3; *p < 0.05, unpaired Student’s t-test). **f** mRNA levels of *Cask*, *GluN1*, *GluN2A*, *GluN2B*, *Kif17*, *Lin7C*, *Abpa1*, and *Nampt* in hippocampi of 20-month-old male mice treated with NMN (300 mg/kg/day) for 1 week. mRNA levels were normalized to those of PBS-treated control mice (*n* = 5; *p < 0.05, unpaired Student’s *t*-test). Data are presented as mean ± SE

To find potential candidate genes which respond to a reduction in hippocampal NAD^+^ levels, we first treated primary hippocampal neurons with FK866 at concentrations of 10 nM and 100 nM for 48 h. Cellular NAD^+^ levels decreased by 60 and 80% at 10 nM and 100 nM of FK866, respectively (Fig. 5a). Nonetheless, there was only a slight decrease in cell viability in the 48-h treatment with 100 nM FK866 (Fig. 5b). Thus, we performed microarray analyses using primary hippocampal neurons treated with 100 nM FK866 for 48 h.^26^ Among genes that responded to cellular NAD^+^ decrease, we found a gene named calcium/calmodulin-dependent serine protein kinase (*Cask*). *Cask* has been reported to be associated with X-linked mental retardation and autism-spectrum disorders.^27,28^ Cask is a multidomain scaffold protein that has been reported to mediate the anterograde transport of vesicles carrying *N*-methyl-d-aspartate receptor (NMDAR) subunits, particularly GluN1 and GluN2B, in neurons.^29^ *Cask* expression in primary hippocampal neurons was reduced by FK866 treatment but restored by adding 100 μM NMN in the presence of FK866 (Fig. 5c). This reduction in *Cask* expression by FK866 was also confirmed in whole hippocampal *ex vivo* cultures (Fig. 5d).

Consistent with our finding that hippocampal NAD^+^ levels decreased over age (Fig. 1a), we found that *Cask* expression also significantly decreased by ~30% between 2 and 19 months of age in the hippocampus (Fig. 5e). We were able to confirm that *Nampt* expression also decreased over age in the hippocampus (Fig. 5e). Remarkably, NMN supplementation in old mice was able to specifically increase *Cask* expression by ~2-fold, whereas the expression of other genes, including NMDAR subunits and vesicle transport subunits, did not show significant changes in response to NMN (Fig. 5f). Therefore, *Cask* expression is regulated in an NAD^+^-dependent manner, and its age-associated reduction can be restored by NMN supplementation *in vivo*.

### SIRT1 regulates *Cask* expression in response to NAD^+^

**Fig. 6:**
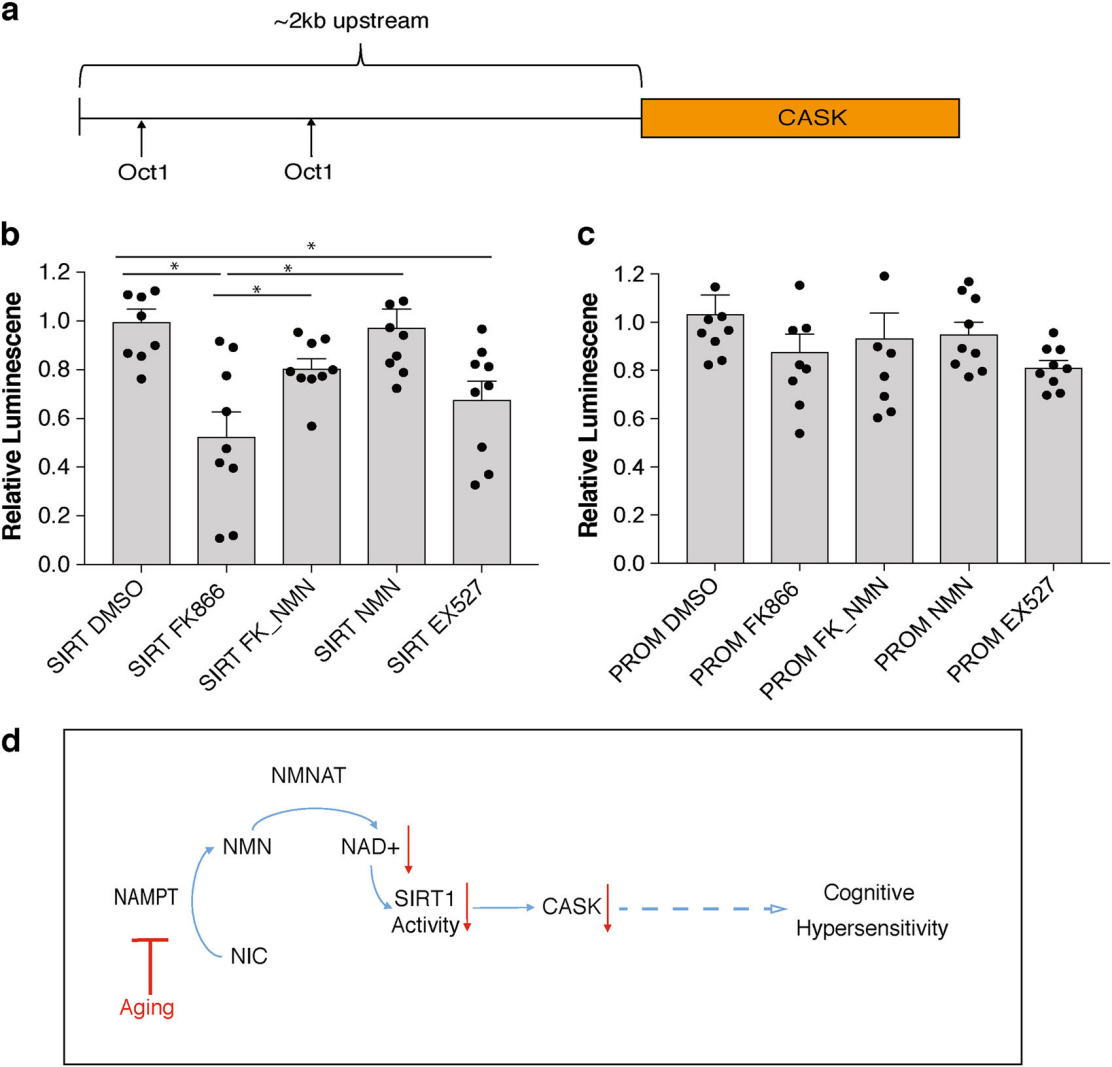
*Cask* promoter activity is regulated in an NAD^+^/SIRT1-dependent manner. **a** The ~2 kb upstream region of the *Cask* gene is schematically shown. There are two potential Oct-1 binding sites. **b**, **c** Relative luciferase activities driven by an ~2 kb *Cask* promoter fragment were measured in HEK293 cells cotransfected with the reporter construct and a *Sirt1* minigene (**b**) or its promoter-only control vector (**c**) with treatment of FK866 (100 nM), FK866 (100 nM) plus NMN (100 μM), NMN (100 μM), or EX527 (10 μM). Luciferase activities were normalized to those of DMSO-treated control cells (*n* = 9; *p < 0.05, one-way ANOVA with LSD post hoc test). Data are presented as mean ± SE. **d** A proposed model for the relationship between age-associated, NAD^+^/SIRT1-mediated reduction in *Cask* expression and cognitive hypersensitivity in old mice

One major group of NAD^+^-dependent mediators is the sirtuin family.^11^ In particular, SIRT1 plays a significant role in regulating hippocampal functions, including long-term potentiation and learning and memory.^12,13^ To examine whether the NAD^+^-dependent regulation of *Cask* is mediated by any sirtuin family member, particularly SIRT1, we employed a transient transfection assay by constructing a luciferase reporter driven by an ~2 kb upstream genomic fragment of the *Cask* gene (Fig. 6a). We used HEK293 cells for this assay because they have very low endogenous expression of the SIRT1 protein. We found that the *Cask* promoter was responsive to changes in NAD^+^ levels when the *Sirt1* minigene was cotransfected^+^ (Fig. 6b). The *Cask* promoter activity was reduced when NAMPT-mediated NAD^+^ biosynthesis was inhibited by FK866, whereas its activity was significantly restored by adding NMN in the presence of FK866. This NAD^+^ dependency of the *Cask* promoter was not observed when no SIRT1 was introduced (Fig. 6c). Additionally, in the presence of SIRT1, the *Cask* promoter activity was significantly suppressed by EX527, a potent SIRT1 inhibitor (Fig. 6b, c), supporting the SIRT1 dependence for the NAD^+^-dependent regulation of the *Cask* promoter. Taken together, *Cask* expression appears to be regulated in an NAD^+^/SIRT1-dependent manner, and it is most likely that age-associated NAD^+^ reduction causes the downregulation of *Cask* expression, possibly contributing to the age-associated changes in hippocampal cognitive functions.

## Discussion

In our present study, we found that older mice developed a cognitive hypersensitivity following exposure to certain aversive environmental stimuli, possibly reflecting age-related alterations in emotionality (fear and/or anxiety) and sensory processing. This hypersensitivity was manifested by greatly increased levels of freezing during tone-shock training, and contextual fear and auditory cue testing. Age-related differences in shock sensitivity may play a role in this heightened freezing response displayed by old mice during the tone-shock training on day 1, which could increase the salience of the foot shock for them and lead to enhanced conditioning, resulting in greatly increased freezing levels during the contextual fear and auditory cue tests. However, increased freezing levels during the altered context baseline measured on day 3 suggest that these differences in freezing to the contextual and auditory cues did not represent genuine conditioning/retention effects. Rather, the old mice became hypersensitive to general environmental changes that occurred during the conditioned fear procedure following exposure to foot shocks. Interestingly, NMN supplementation to old mice was able to mitigate age-associated cognitive hypersensitivity and age-associated increases in shock sensitivity, raising the possibility that modifications in the NMN supplementation procedure (e.g., extended treatment duration) might lead to greater positive effects concerning the putative emotionality component of the age-related hypersensitivity as well.

The age-associated cognitive hypersensitivity described above may be due to hippocampal NAD^+^ decline because the decreased shock sensitivity threshold in the old mice can be ameliorated by short-term NMN supplementation. Moreover, CA1-specific, but not DG-specific, ablation of *Nampt* recapitulated the cognitive hypersensitivity of old mice, providing support for the role of NAMPT-mediated NAD^+^ biosynthesis in hippocampal cognitive functions. We also found that *Cask* expression was regulated in an NAD^+^/SIRT1-dependent manner, and hippocampal *Cask* expression was specifically downregulated in old mice. Among genes whose products are involved in neuronal vesicle transport and synaptic function, only *Cask* showed significant upregulation in old hippocampi in response to short-term NMN supplementation. These results suggest that age-associated reduction in NAMPT-mediated NAD^+^ biosynthesis contributes to the alteration in hippocampus-dependent cognitive functions during aging.

It has been established that NAD^+^ levels are significantly reduced in multiple peripheral tissues, as well as in the brain, during the course of aging.^6^ This reduction in NAD^+^ is, at least in part, due to decreased *Nampt* expression. For example, our previous and current studies have shown that levels of NAD^+^ and *Nampt* decrease with age in the hippocampus.^17^ Interestingly, the ablation of *Nampt* in adult neural stem/progenitor cells (NSPCs) recapitulates their functional defects during aging, and the decrease in the NSPC pool during aging can be rescued significantly by NMN supplementation.^17^ Therefore, loss of both NAMPT and NAD^+^ during aging could evoke significant changes to multiple cognitive and behavioral modalities in mice. Indeed, *CaMKIIαNampt*^−/−^ mice, which specifically lack NAMPT in forebrain excitatory neurons, exhibit a multitude of behavioral and cognitive impairment, including hyperactivity, memory deficit, and reduced anxiety.^22^ Nonetheless, these mice fail to recapitulate the cognitive hypersensitivity detected in old mice. Instead, CA1-specific *Nampt* knockdown mice are able to recapitulate this specific cognitive phenotype of old mice. Whether age-associated NAD^+^ reduction occurs in the CA1 region to a greater extent compared to other hippocampal regions still remains unanswered. It will be of great importance to measure NAD^+^ levels in different hippocampal regions during the course of aging.

Why do old mice develop a cognitive hypersensitivity phenotype? We have identified *Cask* as a potential downstream target in response to age-associated NAD^+^ reduction in the hippocampus. *Cask* expression is sensitive to NAD^+^ changes in hippocampal neurons and *in vivo*. *Cask* expression is significantly downregulated in the hippocampus during aging, which can be ameliorated by NMN supplementation. Cask is a multidomain scaffold protein important in cell junctions and at the synapse.^30^ Specifically, Cask has been reported to interact with the GluN2B subunit of the NMDAR and work in an alternative secretory pathway for the transport of GluN2B-containing vesicles.^29^ Cask deficiency causes an ~40% reduction of NMDARs at the synapse.^29^ Additionally, Cask can potentially be translocated to the nucleus and interact with T-Brain-1 (TBR1), a brain-specific T-box transcription factor.^30–33^ Cask–TBR1 interaction is important to increase the transcriptional activity of TBR1 and upregulate *Grin2b* (*GluN2B*) expression.^32,34–36^ GluN2B is indeed the subunit whose messenger RNA (mRNA) and protein expression shows significant reduction during aging.^37–40^ Thus, age-associated reduction in *Cask* expression could possibly compromise the function of GluN2B-containing NMDARs by affecting its expression levels and/or its transport to the synapse. Given that individuals with mutations in the *Cask* gene develop mental retardation and autism-spectrum disorders,^27,28^ it is conceivable that the age-associated reduction in *Cask* expression could cause a wide range of cognitive impairments through the dysfunction of GluN2B-containing NMDARs. Further investigation will be required to examine whether CA1-specific knockdown of *Cask* or *Grin2b* could recapitulate the cognitive hypersensitivity observed in old mice.

Interestingly, *Cask* expression appears to be regulated in a SIRT1-dependent manner. Because EX527 abrogates the SIRT1-dependent activation of the *Cask* promoter, the NAD^+^-dependent deacetylase activity of SIRT1 is required for this transcriptional regulation. However, the target of SIRT1 for the regulation of the *Cask* promoter remains unknown. We attempted to see whether SIRT1 physically resides on the *Cask* promoter by chromatin immunoprecipitation. However, we have so far failed to detect the existence of SIRT1 on the *Cask* promoter (data not shown). Thus, it is possible that SIRT1 deacetylates a key transcription factor and activates its transcriptional activity for the *Cask* promoter.

Taken together, we propose the following model (Fig. 6d). During the course of normal aging, hippocampal neurons, particularly CA1 neurons, experience a significant decline in NAMPT and NAD^+^ levels. This NAD decrease causes decreased SIRT1 activity, resulting in the reduction in *Cask* expression in the aged hippocampus. This reduction in *Cask* may decrease the localization of GluN2B at the synapse, contributing to the alteration in hippocampal cognitive and behavioral functions during aging. Importantly, NMN, a key NAD^+^ intermediate and a major product of the NAMPT reaction, can ameliorate some aspects of the cognitive hypersensitivity we observed in old mice. If such cognitive hypersensitivity is also observed in the elderly, NMN administration could improve their health conditions and potentially enhance their quality of life.

In conclusion, our study demonstrates that age-associated alteration in cognitive and behavioral functions is induced by reduced NAMPT-mediated NAD^+^ biosynthesis in the hippocampus, particularly in the CA1 region, in old mice. Our study also shows that NMN supplementation, even in the short term, is able to mitigate the age-associated alteration in the sensory processing of some aversive stimuli and possibly other related behaviors. Although further detailed analyses will be necessary, our findings provide critical insights into how aging affects cognitive and behavioral functions and how such impairments can be prevented or treated to enhance the quality of our later lives.

## Methods

### Animals

Mice were maintained ad libitum on a regular chow diet on a 12 h light/dark cycle. All mouse lines were maintained as homozygous colonies. Floxed *Nampt* (*Nampt*^*flox/flox*^) mice^41^ were used for the generation of CA1-specific *Nampt* knockdown mice. In all experiments, control mice were age-matched littermates. Aged C57BL/6 mice and their young controls were provided by the National Institute on Aging. All animal procedures were approved by the Washington University Institutional Animal Care and Use Committee and were in accordance with National Institutes of Health guidelines.

### Cognitive and behavioral assessments

Behavioral tests were conducted in the Animal Behavior Core at Washington University School of Medicine in St. Louis. The assessments for each of the three mouse groups included a battery of sensorimotor measures and 1-h locomotor activity, MWM, and contextual fear conditioning (including shock sensitivity) tests. All tests were performed and analyzed similarly to previously described methods.^22^ Full descriptions of these procedures are provided in the Supplementary Information.

### Stereotactic injection of adeno-associated virus carrying Cre recombinase and GFP genes (AAV-Cre-GFP)

AAV serotype 8 was provided and produced by the Hope Center Viral Vectors Core. Mice were injected with AAV-Cre-GFP or AAV-GFP following the previously described procedure.^42^ Bregma was identified, and appropriate coordinates for the stereotactic injection were registered: relative to Bregma, for the CA1, anterior-posterior (AP) −2 mm, medial-lateral (ML) ±1.5 mm, and dorsal-ventral (DV) −1.7 mm; for the DG, AP −1.5 mm, ML ±1.5 mm, and DV −2 mm. The experimental or control AAV was slowly injected (0.2 μl/min) for a total volume of 1 μl. The incision was closed with 4-0 nylon sutures. Immediately after the injection, animals were given a subcutaneous injection of slow-release buprenephrine as an analgesic and allowed to recover in a temperature-regulated incubator (32 °C) until fully awake, then were transferred to an isolated animal room for 72 h and maintained without disturbance. All injected mice had 4 weeks to fully recover before being used for any experiment.

### NAD^+^ measurement

NAD^+^ levels were determined using an HPLC system (Shimadzu) with a Supelco LC-18-T column (15 × 4.6 cm, Sigma), as described previously.^43^

### Analyses of primary hippocampal neurons

Primary hippocampal neurons were isolated, as previously described.^44^ Briefly, hippocampi were dissected from E16.5–E18.5 C57BL/6J embryos and placed on ice in dissection solution containing Hibernate-E (Gibco). Hippocampi were digested by incubating in 0.25% trypsin-EDTA (Sigma) at 37 °C for 20 min. DNase I was added, and hippocampi were incubated at room temperature for 5 min and washed twice with plating media before final trituration by pipetting in plating media until no clumps remained. Cells were plated onto wells coated with 100 μg/ml poly-l-lysine (Sigma) and treated with 10 μM Ara-C (Sigma) after 2 days in vitro. Primary hippocampal neurons selected in the presence of Ara-C were treated with 10 or 100 nM FK866 or dimethyl sulfoxide (DMSO) and collected and snap-frozen for NAD^+^ measurement and other analyses. Cell viability was assessed using the Pierce LDH Cytotoxicity Assay Kit (Thermo Scientific). Samples were stored at −80 °C until extraction. RNA samples were prepared with the PureLink RNA Mini kit (Ambion) for quantitative real-time reverse transcription-polymerase chain reaction (qRT-PCR).

### Quantitative real-time RT-PCR

RNA was reverse-transcribed into complementary DNA (cDNA) with the High-Capacity cDNA Reverse Transcription Kit (Applied Biosystems). qRT-PCR was conducted with the TaqMan Fast Universal PCR Master mix and appropriate TaqMan primers for each gene in the GeneAmp 7500 fast sequence detection system (Applied Biosystems). For measurement of *Cask* mRNA, a SyBR green probe was used. Primer sequences for *Cask* are: forward 5′-GAAACCAGTTTAGGCATTTGCT-3′; reverse 5′-TGAAATGAAGGACCCAAAGG-3′. Relative expression levels were calculated for each gene by normalizing to GAPDH (glyceraldehyde 3-phosphate dehydrogenase) expression levels and then to a control.

### Laser microdissection of hippocampal subregions

The CA1, CA3, and DG were dissected from AAV-Cre-GFP- or AAV-GFP-injected mice after cognitive and behavioral tests by laser microdissection using the Leica LMD 6000 system (Leica Microsystems, Buffalo Grove, IL, USA). RNA was isolated from each hippocampal subregion using the Arcturus PicoPure RNA Isolation Kit (Life Technologies, Grand Island, NY, USA). As described previously,^45^ for qRT-PCR, each RNA concentration was determined by NanoDrop, and cDNA was synthesized using the Applied Biosystems High-Capacity cDNA Reverse Transcription Kit (Life Technologies, Grand Island, NY, USA).

### Luciferase assay

Luciferase assays were conducted as described previously.^45^ HEK293 cells were transfected with a luciferase reporter driven by an ~2 kb promoter and a *Sirt1* minigene or a control vector that carries only the *Sirt1* promoter.^46^

### Statistical analyses

All data are presented as mean ± SE. Statistical significance between control and experimental samples was determined by unpaired Student’s *t*-test with statistical significance being *p* < 0.05. For the behavioral data, ANOVA models were used. The rmANOVA models containing one between-subject variable (genotype) and one within-subject (repeated measures) variable (e.g., time or sessions) were typically used to analyze the 1 h locomotor activity, MWM, and conditioned fear data. Sex was also included as a between-subject variable for the CA1-specific *Nampt* knockdown study since both males and females were used. The Huynh–Feldt corrected *p* values were provided for all within-subject effects containing more than two levels to help protect against violations of sphericity/compound symmetry assumptions underlying rmANOVA models, although the degrees of freedom were listed for the unadjusted *p* values. Typically, one-way ANOVA models were used to analyze differences between groups for measures in the 1 h locomotor activity and the sensorimotor battery tests and platform crossings and time in the target quadrant during the MWM probe trial. Simple main effect tests and pair-wise comparisons were conducted after relevant, significant overall ANOVA effects were found and were subjected to Bonferroni correction when appropriate. The *p* values equal to 0.0000 are listed as *p* < 0.00005.

## Electronic supplementary material

Supplementary Information

## Data Availability

All data generated or analyzed during this study are included in the article and its Supplementary Information.

## Electronic supplementary material

**Supplementary information** accompanies the paper on the *npj Aging and Mechanisms of Disease* website (10.1038/s41514-018-0029-z).
