# Supplementary material for: CA1 Nampt knockdown recapitulates hippocampal cognitive phenotypes in old mice which nicotinamide mononucleotide improves
Source: NPJ Aging Mech Dis. 2018 Nov 8;4:10. doi: 10.1038/s41514-018-0029-z (PMC6224504; doi:10.1038/s41514-018-0029-z)
Supplement: Supplementary file 1 — Supplementary Information [file 41514_2018_29_MOESM1_ESM.pdf]

Supplementary Information

CA1 *Nampt* Knockdown Recapitulates Hippocampal Cognitive Phenotypes in Old Mice Which Nicotinamide Mononucleotide Improves

Sean Johnson[1,3], David F. Wozniak[2], and Shin-ichiro Imai[1,*]

[1]Department of Developmental Biology,
[2]Department of Psychiatry, The Taylor Family Institute for Innovative Psychiatric Research,
Washington University School of Medicine,
St. Louis, MO 63110, USA
[3]Present address: Department of Gerontology, Laboratory of Molecular Life Science, Institute of Biomedical Research and Innovation, Kobe, Japan

*Co-corresponding authors
Shin-ichiro Imai, M.D., Ph.D.
Professor
Department of Developmental Biology
Department of Medicine (Joint)
Washington University School of Medicine
Campus Box 8103, 660 South Euclid Avenue,
St. Louis, MO 63110, USA
Tel: 314-362-7228 Fax: 314-362-7058 E-mail: imaishin@wustl.edu

Supplementary Results

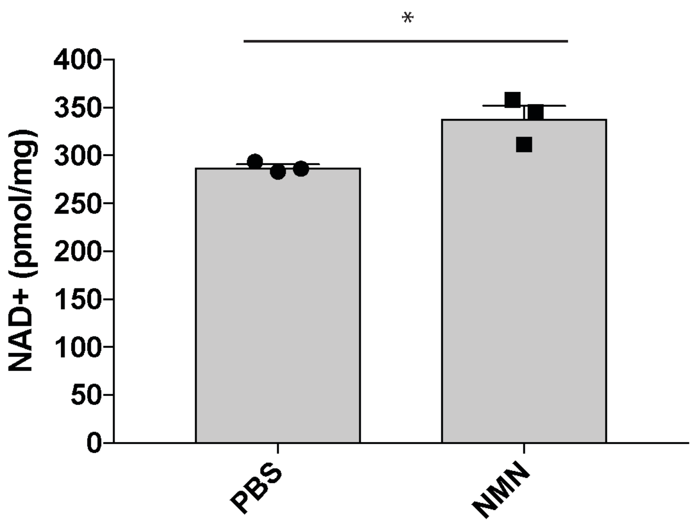

Supplementary Fig 1. NMN administration increases hippocampal NAD^+ levels in 20 month-old mice. NMN was administered via oral gavage at the dose of 300mg/kg to 20 month-old male mice (n=3). (*p<0.05, unpaired Student's t test). Data are presented as mean ± SE.

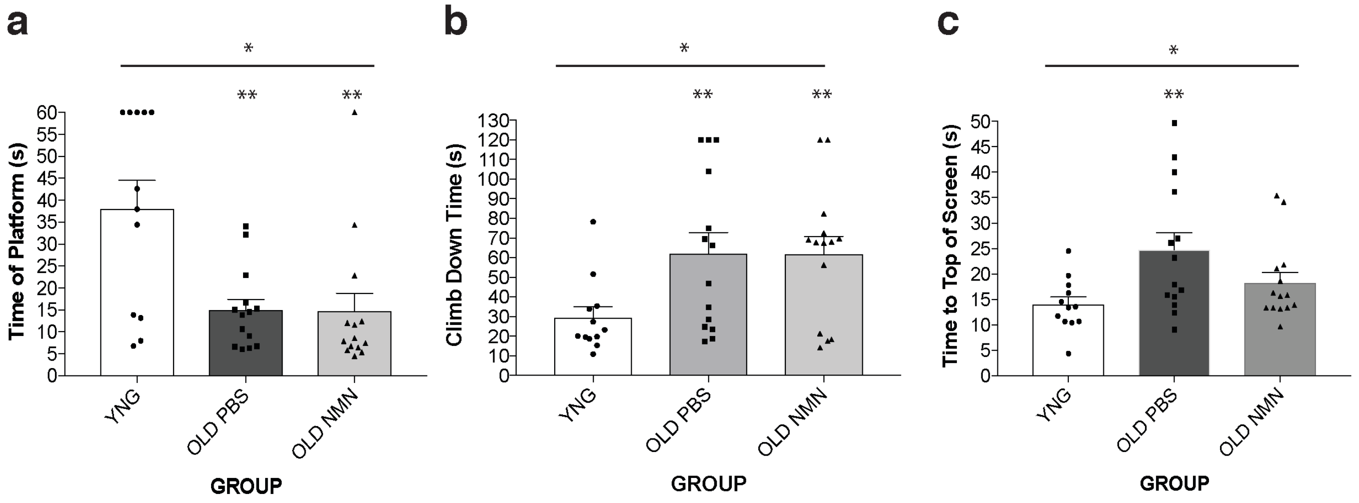

Supplementary Fig 2. Old mice exhibit deficits on sensorimotor tests. Tests were conducted on PBS-treated 2 month-old (YNG; n=12), PBS-treated 20 month-old (OLD PBS; n=14), and NMN-treated 20 month-old (OLD NMN; n=14) male mice. Sensorimotor behaviors were examined. **a,** Aged mice show deficits in time spent on platform. A significant group effect (*p=0.0008) was found regarding performance on the platform test, and subsequent pair-wise comparisons showed that the PBS-treated 20 month-old, and NMN-treated 20 month-old male mice exhibited deficits compared to the PBS-treated 2 month-old group (**p=0.0009 and 0.0007, respectively). **b,** Aged mice exhibited an increase in time required to climb down a pole. Group performance differences on the pole test were observed (group effect: *p=0.025), with significant impairments being found for the PBS-treated 20 month-old and NMN-treated 20 month-old groups relative to the PBS-treated young controls (**p=0.016 and 0.018, respectively). **c,** PBS-treated aged mice showed an increase in time required to climb to the top of a wire mesh screen at an angle of 60°. Group performances differed (group effect: *p=0.018), which were found to be mostly due to significant differences between the PBS-treated young compared to the PBS-treated old mice (**p=0.006). Data are presented as mean ± SE.

Supplementary Methods

Experimental design of behavioral studies

Behavioral tests were conducted on three individual cohorts of naïve mice in three separate studies. In the first study, beginning 4 weeks after stereotactic NMN injections, groups of PBS-treated young (n=12), PBS-treated old (n=14), and NMN-treated old (n=14) male mice, were evaluated on a 1-locomotor activity test, a battery of sensorimotor measures, the Morris water navigation task, and on a conditioned fear test using methods similar to those described previously [1]. The second study involved CA1-specific *Nampt* knockdown mice (n=7; 4M, 3F) and a GFP-injected littermate control group (n=8; 4M, 4F) that were tested on the same behavioral measures. Groups of DG-specific *Nampt* knockdown (n=10) and GFP-littermate control (n=10) male mice were evaluated on these tests in a third study.

1-h locomotor activity and sensorimotor battery

Locomotor activity was evaluated in mice using transparent (47.6 x 25.4 x 20.6 cm high) polystyrene enclosures and computerized photobeam instrumentation as previously described [1]. Total ambulations (whole body movements) was the variable used to characterize general locomotor activity over a 1-h period, which was considered to be the most relevant for evaluating possible confounding influences on freezing behavior quantified during the conditioned fear procedure. The data were analyzed across six 10-min intervals (blocks). The following day the mice were also evaluated on a battery of sensorimotor tests designed to assess balance (ledge and platform), strength (inverted screen), coordination (pole and inclined screens) and initiation of movement (walking initiation), as previously described [1]. For the walking initiation test, a mouse was placed on a surface in the center of a 21 x 21 cm square marked with tape and the time it took for it to exit the square was recorded. During the balance tests, the time was recorded that the mouse remained on a Plexiglas ledge (0.75 cm wide) or a small circular wooden platform (3.0 cm in diameter) elevated 30 cm or 47 cm, respectively. The screen tests were conducted by placing a mouse head-oriented down in the middle a wire mesh grid measuring 16 squares per 10 cm, elevated 47 cm and angled

at 60° or 90°. The time was recorded that the mouse required to turn 180° and climb to the top of the apparatus. For the inverted screen, the mouse was placed on the 60° screen as described above and once it appeared to have a secure grip, the screen was inverted 180° and the time the mouse remained on to the screen was recorded. Each test had a maximum time of 60 s, except for the pole test, which had a maximum time of 120 s. The averaged time of two trials for each test was used for the analyses.

Morris water maze test

Spatial learning and memory were evaluated in the first cohort of mice on Morris water maze (MWM) task using a computerized tracking system (ANY-maze, Stoelting Co., Wood Dale, Il) as previously described [1] on the day following the completion of the testing on the sensorimotor battery. Cued (visible platform, variable location) and place (submerged, hidden platform, constant location) trials were conducted and escape path length, latency, and swimming speeds were computed. The cued condition involved conducting four trials per day (60 s maximum per trial) for two consecutive days with the platform being moved to a different location for each trial using a 30-min inter-trial interval (ITI), and with very few distal spatial cues being present to limit spatial learning. Performance was analyzed across four sessions where each session consisted of one block of two trials with two sessions being conducted on each of two days of testing for a total of four sessions. Three days later, place trials were initiated to assess spatial learning where mice were required to learn the single location of a submerged platform in the presence of several salient distal spatial cues. During place trials, the mice received five daily test sessions where each session consisted of two blocks of two consecutive trials [60 s maximum for a trial; 30-s ITI (spent on platform)] with each block being separated by approximately 2 h and each mouse being released from a different quadrant for each trial. The place trials data were analyzed over five sessions (four trials/session) where each session represented the performance level for each of five consecutive days. Retention performance was assessed during a probe trial (platform removed; 60-sec maximum) that was administered approximately 1 h after the last place trial on the 5th day of training with the platform being removed and the mouse being released

from the quadrant opposite to where the platform had been located. Time spent in the various pool quadrants including the target quadrant where the platform had been placed and crossings over the exact platform location served as the dependent variables.

Conditioned Fear test

Mice were also tested on a conditioned fear procedure, as previously described [1], following completion of the MWM testing. Briefly, the procedure involved training and testing the mice in two Plexiglas conditioning chambers (26 cm x 18 cm, and 18 cm high) (Med-Associates, St. Albans, VT) with each chamber containing distinct and different visual, odor, and tactile cues. On day 1, each mouse was placed into the conditioning chamber for a 5-min trial and freezing behavior was quantified during a 2-min baseline period. Beginning at 3 min and at 60-s intervals thereafter, the mice were exposed to 3 tone-shock pairings where each pairing included a 20-s presentation of an 80 dB tone (conditioned stimulus; CS) consisting of broadband white noise followed by a 1.0 mA continuous footshock (unconditioned stimulus; UCS) presented during the last second of the tone. Broadband white noise was used instead of a frequency-specific tone in an effort to avoid possible auditory deficits that might occur with age. The mice were placed back into the conditioning chamber the following day and freezing behavior (conditioned response; CR) was quantified over an 8-min period to evaluate contextual fear conditioning. Twenty-four hours later, the mice were placed into the other chamber containing different cues and freezing behavior was quantified during a 2-min "altered context" baseline and over the subsequent 8 min, during which time the auditory cue (tone; CS) was presented. Freezing was quantified using *FreezeFrame* image analysis software (Actimetrics, Evanston, IL), which allowed for simultaneous visualization of behavior while adjusting a “freezing threshold,” which categorized behavior as freezing or not freezing during 0.75 s intervals. Freezing was defined as no movement except for that associated with normal respiration, and the data were presented as percent of time spent freezing.

Shock Sensitivity

Sensitivity to foot shock was evaluated following completion of the conditioned fear testing, using a modification of a procedure that has been described previously [2]. Shock sensitivity testing was performed 24-72h after the fear conditioning procedure was completed. Mice were placed in the conditioning chambers for two minutes and then exposed to 1 s shocks every 20–30 s in order of increasing intensity (shocks started at 0.1 mA and were increased by 0.05 mA increments in intensity). The level of shock required to elicit flinching, vocalization or escape-related behavior was determined through behavioral observations following shock onset. Flinching was defined when a mouse displayed any of the following somatic/postural changes after shock onset: abruptly stops moving; backs up slightly; turns head toward the grid floor; displays eye squinting/blinking. Escape-related behavior was judged to occur when the following behaviors were observed subsequent to the onset of shock: rapid walking or backing up; darting; jumping; running; frantic side-to-side movements.

References

1 Stein, L. R. et al. Expression of Nampt in hippocampal and cortical excitatory neurons is critical for cognitive function. J. Neurosci. 34, 5800-5815, doi:10.1523/JNEUROSCI.4730-13.2014 (2014).

2 Khuchua, Z. et al. Deletion of the N-terminus of murine map2 by gene targeting disrupts hippocampal ca1 neuron architecture and alters contextual memory. Neuroscience 119, 101-111 (2003).
